# New Appliances and Things Medical

**Published:** 1900-09-15

**Authors:** 


### NEW APPLIANCES AND THINGS MEDICAL.

**[We shall be glad to receive, at our Office, 28 & 29, Southampton Street, Strand, London, W.C., from the manufacturers, specimens of all new preparations and appliances which may be brought out fromti me to time.]**

### SOMATOSE.

(BRITISH SOMATOSE COMPANY, LTD., 165, QUEEN VICTORIA STREET, LONDON, E.C.)

SOMATOSE is a predigested and desiccated preparation of albumen of meat. It contains something like 50 per cent. of deutero-albumose and 13 per cent. hetero-albumose, and a small quantity of peptone and mineral matter. Just as some years ago peptones replaced the formerly popular meat extracts in the treatment of wasting diseases, so now once more physiology teaches us that our lately acquired knowledge is erroneous and that peptones are of considerably lower physiological value as food stuffs than they were supposed to be, and that they are only partially or not at all metabolised into living tissues. The albumoses on the other hand are now regarded as proximate principles of true nutritive value, capable of supplying energy and repairing tissue waste. Somatose is almost tasteless, it readily dissolves in water, causes no gastric irritation, and does not lead to the presence of albumen or peptone in the urine. In those cases in which there is interference with the powers of digestion and assimilation, as in gastritis, gastric ulcer and disease of the intestine, Somatose has proved to be of the greatest value, and the only objection to its general adoption in all such cases appears to be the expense. There are now two other forms of Somatose, namely, Iron and Milk Somatose, the former supplying an assimilable form of iron food and of value in anæmia, and the latter, which is combined with a certain proportion of tannic acid owing to its mildly astringent properties, being specially indicated in cases of chronic diarrhœa and atonic condition of the bowels.

### "SHELL BRAND" FLOOR POLISH.

(HAMILTON AND CO., 106, ELLIOT STREET, GLASGOW.)

THE "Shell Brand" floor polish is a preparation of most careful manufacture, invaluable for removing grease and dirt, and imparting a very fine surface to parquet and stained floorings. To a certain extent it acts as a preservative of the wood by filling up the pores and crevices with a deodorant and slightly antiseptic wax. It has a pleasant, aromatic odour, and is altogether a cleanly and efficacious medium for the polishing of wax cloths, linoleum, and wooden floors.

